# Approaches to Minimizing Periprocedural Complications During Subcutaneous Implantable Cardioverter-defibrillator Placement

**DOI:** 10.19102/icrm.2020.110504

**Published:** 2020-05-15

**Authors:** Srikanth Vedachalam, Schuyler Cook, Tanner Koppert, Toshimasa Okabe, Raul Weiss, Muhammad R. Afzal

**Affiliations:** ^1^Division of Cardiovascular Medicine, Wexner Medical Center, Ohio State University Medical Center, Columbus, OH, USA; ^2^Department of Internal Medicine, Adena Regional Medical Center, Chillicothe, OH, USA

**Keywords:** Complications, periprocedural, subcutaneous implantable cardioverter-defibrillator

## Abstract

The subcutaneous implantable cardioverter-defibrillator (S-ICD) is the latest option among devices clinically available for the prevention of sudden cardiac death, with experience from previous trials and postmarketing studies supporting the feasibility and safety of this kind of system. The extracardiac positioning of the S-ICD obviates the need for transvenous leads, which translates into lower incidence rates of lead-related complications and systemic infections. This review will highlight the results of pertinent studies related to the perioperative management of S-ICDs and review potential approaches to minimizing the risk of complications such as hematoma at the pulse generator location, unsuccessful defibrillation due to suboptimal S-ICD lead and generator positioning, and postoperative pain. An extensive literature search using PubMed was conducted to identify relevant articles.

## Introduction

The subcutaneous implantable cardioverter-defibrillator (S-ICD) is the latest device option in the area of defibrillation therapy for the prevention of sudden cardiac death (SCD).^1–4^ Several studies have shown that the efficacy of S-ICDs is comparable to that of transvenous devices in the successful defibrillation of ventricular arrhythmias.^5–7^ Owing to the S-ICD’s extravascular implantation profile and lack of transvenous leads, the risks of both short-term and long-term complications are lower than as associated with transvenous devices.^8,9^ Using the extravascular location is of particular advantage in patients with limited venous access, a higher risk of infection such as those with left ventricular assist devices (LVADs), a history of intravenous drug use, prior transvenous device infection, and end-stage renal disease on dialysis.^10–12^

During the early experience with S-ICDs, there was an increased incidence of inappropriate shock (IAS) resulting primarily from T-wave oversensing (TWOS).^11^ With preoperative screening electrocardiography (ECG) and the incorporation of novel sensing algorithms, the incidence of IAS has been significantly reduced.^12^ Over time, there have been several advances made in the perioperative management of S-ICDs, with further evolution continuing to occur. In the contemporary experience, many implanters routinely conduct intermuscular S-ICD implantation via the two-incision approach.^13^ The S-ICD is a safe and effective device modality for both primary and secondary prevention of SCD and may support a shorter length of hospital stay.

This review sought to elaborate the various perioperative steps able to minimize the risk of complications during S-ICD implantation. To this end, the perioperative steps have been categorized into preoperative, intraoperative, and postoperative components.

## Preoperative considerations

### Appropriate patient selection

Preoperative screening ECG is the single most important step in assessing the eligibility for S-ICD. Appropriate patient selection results in the reduction of IAS due to TWOS. During screening, ECG recordings are obtained using limb lead electrodes to simulate the S-ICD–sensing vectors. The resulting leads (leads I, II, and aVF) correspond to the primary, secondary, and alternate vectors of S-ICD, respectively. Screening ECGs are analyzed using the template of the patient screening tool provided by the manufacturer of the S-ICD (Boston Scientific, Natick, MA, USA). The screening tool takes into account the relative amplitude and duration of T-wave and QRS. At least one vector is required to qualify for the S-ICD. Recently, an automated screening tool has also been introduced. Adequate screening ensures appropriate sensing and minimizes inappropriate therapies resulting from TWOS. This is achieved by using a score system that is primarily based on the QRS signal amplitude and the signal ratio of the QRS complex to T-wave. Although routine preoperative screening ECGs and improvements in existing discrimination algorithms have resulted in significant reductions in the incidence of IAS, oversensing issues continue to persist.^14^

### Subcutaneous implantable cardioverter-defibrillator placement in patients with higher body mass indices

**Figure 1:**
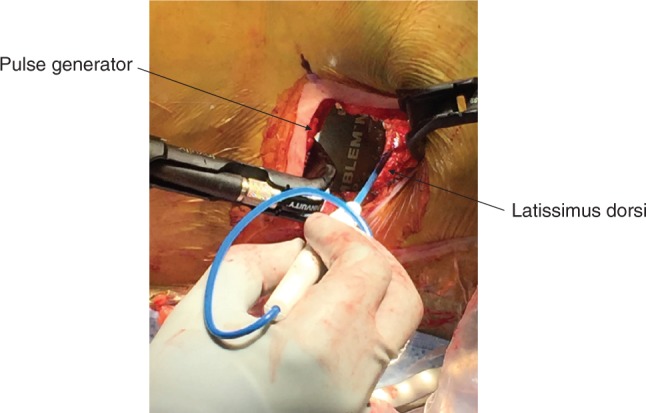
Proper positioning of the S-ICD pulse generator in the lateral pocket.

Previous studies have suggested that patients with higher body mass indices (BMI) of more than 30 kg/m^2^ experience increased incidence rates of IAS and failed defibrillation testing (DT) and a greater failure rate of defibrillation after implant.^15,16^ However, more recent studies indicate that appropriate positioning of the generator and sternal electrode minimizes the impact of BMI.^9,15,16^ According to Amin et al.,^15^ the positioning of the pulse generator and electrode is “optimal” if the pulse generator is not inferior to the sixth intercostal space, the electrode is not inferior to the xiphoid process, and the distance between the electrode coil and the sternum is less than 3 mm. In this optimal system positioning, a similar success rate of first shock was seen between individuals with BMI values of more than 30 kg/m^2^ and less 30 kg/m^2^ (95.2% versus 90.5%, respectively).^15^ When a patient with a higher BMI is deemed an appropriate candidate for S-ICD placement, it should be ensured that the pulse generator is located in the intermuscular space between the latissimus dorsi and the serratus anterior **(Figure 1)** and adequately anchored to the chest wall with two anchoring sutures. The pulse generator should be located along the midaxillary line within the fascial plane.^15^ The electrode should travel closely along the left parasternal space. If not properly placed, patients are at risk for higher impedance and failed shock. The most common cause of suboptimal device location in obese individuals is the tendency to place the device too superficially within the subcutaneous fat.^15^

### Preprocedural marking under fluoroscopy

Many electrophysiologists implant S-ICDs without the use of intraoperative fluoroscopy. It is advisable, however, to mark the intended position of the electrode and generator before skin preparation using a sample electrode and generator guided by fluoroscopy, particularly when new S-ICD implanters gain experience with the optimal implanting technique. Preprocedural identification of the optimal system position can reduce unnecessary incisions in the suboptimal locations, leading to neurovascular damage and perioperative complications. The lateral pocket is especially prone to higher events of bleeding and hematoma formation, given its vascular-rich environment and lack of tamponade effect.^17^

## Intraoperative consideration

### Choice of anesthesia

Initially, all S-ICD implantations were performed using general anesthesia. However, as the operators gained more experience and achieved shorter procedure durations, the use of monitored anesthesia care (MAC) began to increase. In a single-center experience, S-ICD implantation was safe and feasible with MAC.^18^ The use of MAC is anticipated to decrease complications associated with endotracheal intubation and prevent significant hemodynamic compromise associated with general anesthesia. Sufficient analgesic control can be facilitated with intermittent intravenous boluses of analgesia during critical S-ICD implanting steps, such as pocket creation, lead tunneling, and DT. A retrospective study from Ohio State University has suggested that S-ICD implantation is feasible and safe with MAC **(Table 1)**.^19^

### Device implantation

**Figure 2:**
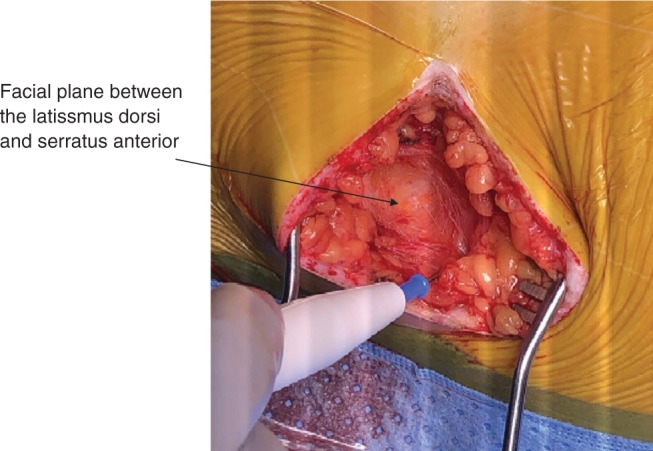
Identification of the fascial plane between the latissimus dorsi and the serratus anterior.

Establishing appropriate analgesia is critical before making the initial incision. Owing to the larger size of the pulse generator, extensive dissection is required in the lateral pocket. At our institution, bupivacaine infiltration (maximum dose: 2.5 mg/kg or 175 mg/dose) is used instead of lidocaine at the incision sites. Bupivacaine has a longer half-life of between two and three hours, and its analgesic effect lasts up to 14 hours. Meanwhile, bupivacaine has a slower onset of action and the implanter should allow for at least five minutes to pass after the skin is infiltrated with the local anesthetic. The two-incision approach has become a standard for S-ICD implantation. The first incision is made along the fourth to sixth intercostal space to create a lateral pocket for the pulse generator. The second incision located in the subxiphoid area should be made at the lower edge of the sternum. Following exposure of the lateral pocket, the device should be inserted in the intermuscular area between the latissimus dorsi and the serratus anterior **(Figure 1)**. Identification and blunt dissection to the intermuscular plane are critical to facilitate proper posterior generator location, reduce shock impendence, and establish a durable anchoring site. After skin incision, electrocautery should be used to access the fibrous fascial plane located superficially to the serratus anterior in a lateral and posterior fashion **(Figure 2)**. Once the fibrous area is reached, gentle blunt dissection with a sweeping finger in this fascial plane can create a pocket between the two muscles.

**Figure 3:**
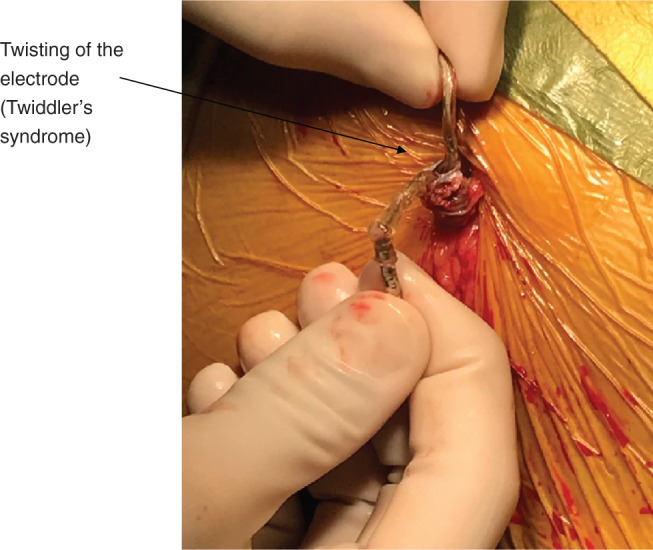
Twisting of the electrode, resulting in Twiddler’s syndrome.

It should be kept in mind that, during extension of the incision with electrocautery, the operator may encounter muscular fibers that are present in the subcutaneous area. These muscle fibers can be mistaken for the latissimus dorsi, prompting the operator to start lateral and posterior dissection. However, if a pocket is created in this area, it may cause significant bleeding due to the presence of a rich vascular plexus. Once proper placement is achieved, the S-ICD pulse generator should be appropriately sutured at two locations. This will minimize device migration and rotation with normal daily activities, particularly when the patient is lying on the left side, which can affect vector sensing by lowering the amplitudes of the QRS complexes.^14^ Suturing at two locations also lowers the risk of twisting the electrode **(Figure 3)**. Proper implantation of the sternal electrode depends on the operator maintaining close proximity between the tunneling tool and the sternum to ensure adequate location during the tunneling process. Tunneling typically starts at the subxiphoid pocket and is advanced toward the lateral pocket. The electrode should not be inferior to the xiphoid process or should only be one rib space below the last sternal wire in those without xiphoid processes due to the previous sternal procedures.^15^

### Use of lighted electrocautery device or retractor

**Figure 4:**
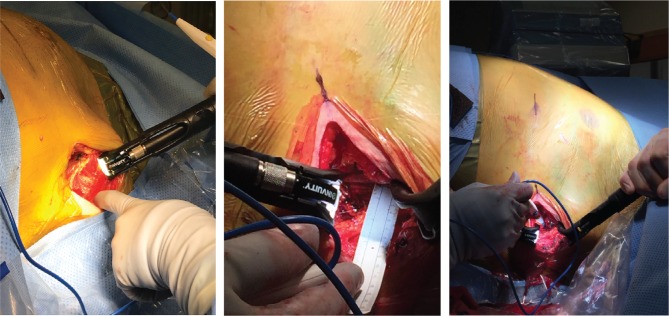
Demonstrations of lighted retractor application to illuminate the lateral pocket.

Because of the large size of the space between the latissimus dorsi and the serratus anterior, adequate visualization of the entire pocket may be challenging. This may hinder achieving adequate hemostasis in this highly vascular area. Previous studies have shown that clinically significant hematomas could develop in this pocket, particularly in patients on uninterrupted anticoagulation.^17,20^ This becomes particularly important in patients with higher BMI values who have deeper lateral pockets. In these situations, a lighted retractor or an electrocautery device with a light source can be useful in ensuring adequate visualization of the pocket to achieve hemostasis **(Figure 4)**.

### Perioperative management of anticoagulation

Some patients undergoing S-ICD implantation may have other comorbidities necessitating uninterrupted or minimally interrupted anticoagulation and antiplatelet medication regimens, such as coronary artery disease, atrial fibrillation, mechanical valve prostheses, intracardiac thrombus, and left ventricular assist device (LVAD) use. Previous studies have shown that uninterrupted anticoagulation during S-ICD implantation increases the risk of postoperative hematomas.^17,20,21^ Those studies suggest that, if there are no contraindications, warfarin should be withheld to allow normalization of the international normalized ratio prior to the procedure. Generally, direct oral anticoagulants (DOACs) should be held for 24 hours before the procedure. There are limited data available regarding the use of DOACs in the perioperative period for S-ICD; to our knowledge, there is only one study available that included two patients on DOACs undergoing S-ICD implant.^21^ DOACs were not withheld in these patients prior to S-ICD implantation and there was no hematoma at the pulse generator site. There was also no increased incidence of hematoma with uninterrupted dual antiplatelet therapy in this study.

### Defibrillation testing

DT is recommended at the time of initial S-ICD implantation and it is our routine practice to conduct DT unless contraindicated.^22^ However, despite the class I recommendation, the National Cardiovascular Data Registry showed that approximately 25% of S-ICD recipients between 2012 and 2015 did not undergo DT.^23^ Further, there is a growing body of evidence suggesting that forgoing DT after S-ICD implantation may be appropriate.^24,25^ Larger prospective studies are needed to confirm the safety of forgoing DT in S-ICD procedures. The ongoing Randomized Trial of S-ICD Implantation with and without DT (PRAETORIAN-DFT) study is seeking to evaluate the value of DT based on postimplant chest X-ray parameters.^26^ A 10-J shock can also be used to determine impedance in patients where the full energy shock is contraindicated. Adequate impedance is probably the most important determinant of successful procedural and clinical defibrillation.

## Postoperative consideration

### Postoperative pain control

Severe pain immediately after new S-ICD implantation is common and may last for three days to five days. Prophylactic pain control measures can be taken to minimize postoperative pain. The authors have introduced a novel pain management protocol referred to as the DASH (Same-day S-ICD and Send Home) strategy that involves the oral administration of a single dose of Tylenol 975 mg (Johnson & Johnson, New Brunswick, NJ, USA) and a single dose of oxycodone 10 mg before the procedure, 0.25% bupivacaine for the incision, and a short supply of Percocet 5/325 mg (Endo Pharmaceuticals, Dublin, Ireland) every six hours or as needed for 48 hours after the procedure in addition to the conduct of nurse-led follow-up phone calls. Overall, this strategy has proven to be beneficial for adequately controlling postoperative pain. Thus, it can be suggested that adequate perioperative pain control facilitates same-day discharge after S-ICD implantation.

### Role of exercise electrocardiograms in minimizing the risk of T-wave oversensing

During the initial experience with S-ICDs, the incidence of IAS was significantly higher when compared with during the contemporary experience.^27^ Previously, exercise ECG testing was routinely performed postimplantation in an effort to optimize the sensing vector and to reduce the risk of oversensing and inappropriate therapy. A retrospective study from our group showed that routine postoperative exercise ECG testing was not necessary to reduce TWOS.^11^ The use of dual-zone programming (addition of conditional shock zone for supraventricular tachycardia discrimination) and the proprietary SMART PASS algorithm (Boston Scientific, Natick, MA, USA) has been shown to reduce IAS and should be routinely implemented in newly implanted devices.^12,14^

### Interaction with left ventricular assist device

A large multicenter series has suggested that concomitant S-ICD implantation and LVAD is feasible. There is a small risk of inappropriate therapy due to electromagnetic interference from the LVAD that can be circumvented by changing the sensing vector of the S-ICD after LVAD placement. Only one patient from the series required subsequent transvenous ICD placement due to electromagnetic interference leading to sensing failure in all three vectors.^10^

## Conclusions

Increases in the sophistication of arrhythmia detection methods and the evolution of novel surgical techniques for proper S-ICD placement have contributed to more effective and safer S-ICD applications for SCD prevention. An effective prophylactic analgesic protocol and the widespread use of MAC for S-ICD implantation may support routine same-day discharge.

### Expert opinion

S-ICD has emerged as a viable alternative to transvenous defibrillators for the prevention of SCD in patients without a pacing indication or a known history of monomorphic ventricular tachycardia amenable to antitachycardia pacing. Multiple studies have proven that the shock efficacy of S-ICD is equivalent to that of transvenous devices. Generally, S-ICD poses no to only a minimal risk of severe infection as a result of the lack of intravascular lead. DT should be routinely performed to ensure appropriate sensing and defibrillation of induced ventricular fibrillation. Over the years, S-ICD implantation techniques have significantly evolved. Many implanters conduct intermuscular implantation using the two-incision technique. The use of MAC is becoming a common practice. Optimal perioperative care includes withholding anticoagulation prior to implantation, prophylactic preprocedural administration of oral analgesic medications, intraoperative use of long-acting local anesthetics (eg, bupivacaine), and a short course of narcotics as needed. These strategies have enabled S-ICD implantation to done on an outpatient basis. Some of the key considerations for S-ICD implant include preimplantation screening using manual or automated ECG screening tools, identifying body surface landmarks using preprocedural fluoroscopy, creating an intermuscular pocket for the pulse generator, and placement of the lead electrode close to the sternum. The relatively high incidence rate of IAS noted during early experience has been significantly reduced in recent years, primarily due to the conduct of preprocedural ECG screening and the incorporation of advanced sensing algorithms. There is a growing body of literature to suggest that foregoing DT may be safe; however, confirmation of such a fact requires further research.^24,25^ Combining S-ICDs and leadless pacing systems provides bradycardia and antitachycardia pacing and may enhance arrhythmia detection and discrimination. The success of such an arrangement would completely eliminate the need for transvenous leads in the majority of patients.

## Figures and Tables

**Table 1:** MAC Versus General Anesthesia in S-ICD Placement

Clinical Characteristics	MAC (n = 111)	General Anesthesia (n = 176)	p-value
Age, mean ± SD	54 ± 14 years	48 ± 13 years	0.002
BMI, mean ± SD	29 ± 7	32 ± 7	0.006
LVEF, mean ± SD	30 ± 7	36 ± 16	0.003
Procedure success, %	100%	99%	NA
Transition to intubation	0	NA	NA
Procedure duration, mean ± SD	78 ± 28 min	83 ± 36 min	0.2
Time in the PACU, mean ± SD	63 ± 39 min	69 ± 39 min	0.2
Same-day discharge, n/n (%)	38/107 (36%)	26/149 (14%)	0.0001

MAC: monitored anesthesia care; SD: standard deviation; BMI: body mass index; LVEF: left ventricular ejection fraction; PACU: postanesthesia care unit.

## References

[1] Kamp NJ, Al-Khatib SM (2019). The subcutaneous implantable cardioverter-defibrillator in review.. Am Heart J..

[2] Pfenniger A, Knight BP (2019). Evolution of extravascular implantable defibrillator technologies.. Prog Cardiovasc Dis..

[3] Santini M, Cappato R, Andresen D (2009). Current state of knowledge and experts’ perspective on the subcutaneous implantable cardioverter-defibrillator.. J Interv Card Electrophysiol..

[4] van Dijk VF, Boersma LV (2019). The subcutaneous implantable cardioverter defibrillator in 2019 and beyond.. Trends Cardiovas Med..

[5] Burke MC, Gold MR, Knight BP (2015). Safety and efficacy of the totally subcutaneous implantable defibrillator: 2-year results from a pooled analysis of the IDE study and EFFORTLESS registry.. J Am Coll Cardiol..

[6] Lambiase PD, Barr C, Theuns DA (2014). Worldwide experience with a totally subcutaneous implantable defibrillator: early results from the EFFORTLESS S-ICD registry.. Eur Heart J..

[7] Weiss R, Knight BP, Gold MR (2013). Safety and efficacy of a totally subcutaneous implantable-cardioverter defibrillator.. Circulation.

[8] El-Chami MF, Levy M, Kelli HM (2015). Outcome of subcutaneous implantable cardioverter defibrillator implantation in patients with end-stage renal disease on dialysis.. J Cardiovasc Electrophysiol.

[9] Boersma LV, El-Chami MF, Bongiorni MG (2019). Understanding outcomes with the EMBLEM S-ICD in primary prevention patients with low EF study (UNTOUCHED): clinical characteristics and perioperative results.. Heart Rhythm..

[10] Afzal MR, Ahmed A, Prutkin JM (2018). Multicenter experience of concomitant use of left ventricular assist devices and subcutaneous implantable cardioverter-defibrillators.. JACC Clin Electrophysiol..

[11] Afzal MR, Evenson C, Badin A (2017). Role of exercise electrocardiogram to screen for T-wave oversensing after implantation of subcutaneous implantable cardioverter-defibrillator.. Heart Rhythm.

[12] Kaya E, Rassaf T, Wakili R (2019). Subcutaneous ICD: current standards and future perspective.. Int J Cardiol Heart Vasc.

[13] Knops RE, Olde Nordkamp LR, de Groot JR, Wilde AA (2013). Two-incision technique for implantation of the subcutaneous implantable cardioverter-defibrillator.. Heart Rhythm.

[14] Afzal MR, Badin A, Weiss R, Augostini R, Hummel JD (2017). T-wave oversensing from postural changes: a rare cause of inappropriate shock from a subcutaneous defibrillator.. HeartRhythm Case Rep.

[15] Amin AK, Gold MR, Burke MC (2019). Factors associated with high-voltage impedance and subcutaneous implantable defibrillator ventricular fibrillation conversion success.. Circ Arrhythm Electrophysiol.

[16] Frankel DS, Burke MC, Callans DJ, Stivland TM, Duffy E, Epstein AE (2018). Impact of body mass index on safety and efficacy of the subcutaneous implantable cardioverter-defibrillator.. JACC Clin Electrophysiol..

[17] Afzal MR, Mehta D, Evenson C (2018). Perioperative management of oral anticoagulation in patients undergoing implantation of subcutaneous implantable cardioverter-defibrillator.. Heart Rhythm..

[18] Essandoh MK, Otey AJ, Abdel-Rasoul M (2016). Monitored anesthesia care for subcutaneous cardioverter-defibrillator implantation: a single-center experience.. J Cardiothorac Vasc Anesth..

[19] Afzal MR, Okabe T, Koppert T (2019). Implantation of subcutaneous defibrillator is feasible and safe with monitored anesthesia care.. Pacing Clin Electrophysiol.

[20] Evenson C, Saour B, Afzal MR, Knight B, Okabe T, Weiss R (2019). Increased risk of hematoma with uninterrupted warfarin in patients undergoing implantation of subcutaneous implantable cardioverter defibrillator.. Pacing Clin Electrophysiol..

[21] Sheldon SH, Cunnane R, Lavu M (2018). Perioperative hematoma with subcutaneous ICD implantation: impact of anticoagulation and antiplatelet therapies.. Pacing Clin Electrophysiol.

[22] Wilkoff BL, Fauchier L, Stiles MK (2016). 2015 HRS/EHRA/APHRS/SOLAECE expert consensus statement on optimal implantable cardioverter-defibrillator programming and testing.. Europace..

[23] Friedman DJ, Parzynski CS, Heist EK (2018). Ventricular fibrillation conversion testing after implantation of a subcutaneous implantable cardioverter defibrillator: report from the national cardiovascular data registry.. Circulation.

[24] Peddareddy L, Merchant FM, Leon AR, Smith P, Patel A, El-Chami MF (2018). Effect of defibrillation threshold testing on effectiveness of the subcutaneous implantable cardioverter defibrillator.. Pacing Clin Electrophysiol..

[25] Miller MA, Palaniswamy C, Dukkipati SR (2017). Subcutaneous implantable cardioverter-defibrillator implantation without defibrillation testing.. J Am Coll Cardiol..

[26] Quast ABE, Baalman SWE, Betts TR (2019). Rationale and design of the PRAETORIAN-DFT trial: a prospective randomized Comparative trial of subcutaneous implantable cardioverter-defibrillator implantation with and without defibrillation testing.. Am Heart J.

[27] Gold MR, Weiss R, Theuns DA (2014). Use of a discrimination algorithm to reduce inappropriate shocks with a subcutaneous implantable cardioverter-defibrillator.. Heart Rhythm.

